# Causes of death and types of injuries of avalanche fatalities based on forensic data: a scoping review

**DOI:** 10.1016/j.resplu.2025.101101

**Published:** 2025-09-13

**Authors:** Céliane Romy, David Eidenbenz, Silke Grabherr, Ken Zafren, Cécile Jaques, Nicolas Hall, Mathieu Pasquier

**Affiliations:** a University of Lausanne, Lausanne, Switzerland; b Department of Emergency Medicine, Lausanne University Hospital, Switzerland; c University Center of Legal Medicine, Lausanne - Geneva, Switzerland, Lausanne University Hospital and University of Lausanne, Geneva University Hospital and University of Geneva, Switzerland; d Department of Emergency Medicine, Stanford University Medical Center, Stanford, California, USA. Alaska Native Medical Center, 10181 Curvi Street, Anchorage, AK 99507, USA; e Lausanne University Medical Library, Lausanne, Switzerland; f Department of Emergency Medicine, Lausanne University Hospital and University of Lausanne, Switzerland; g Department of Emergency Medicine, Lausanne University Hospital and University of Lausanne, rue du Bugnon 46, 1011 Lausanne, Switzerland

**Keywords:** Autopsy, Avalanche, Histopathology, Imaging, Injury, Postmortem

## Abstract

**Background:**

The main causes of death in avalanche victims are asphyxia, trauma, and hypothermia. However, most evidence is based on epidemiological studies with inconsistent forensic data. We aimed to integrate current evidence on causes of death and types of injuries in avalanche victims undergoing postmortem examination.

**Methods:**

We conducted a scoping review of studies reporting forensic data on avalanche-related deaths. Eligible studies included victims who underwent postmortem examination, excluding those buried in buildings or vehicles. Extracted data included study and population characteristics, postmortem investigation (external examination, autopsy, histopathology, toxicology, and imaging), and results (causes of death, types of injuries).

**Results:**

We included 38 studies, of which 31 reported original data involving 1543 fatalities. Of these, 862 (56 %) underwent postmortem examination, with 442 (51 %) receiving autopsies. Among 387 autopsied cases with reported causes of death, asphyxia accounted for 72 %, trauma 18 %, hypothermia 2 %, and combined causes 8 %. Asphyxia-related findings included pulmonary edema, organ congestion, and petechiae. Trauma-related deaths involved head, neck, and thoracic injuries. Hypothermia findings included Wischnewski spots and frostbite. Histopathology (n = 48) revealed asphyxia-related lesions in all victims, while hypothermia findings were infrequent. Postmortem imaging (n = 12) showed pulmonary edema. Toxicology detected ethanol in 4 %, cannabinoids in 11 % and cocaine in 1 % of cases tested.

**Conclusion:**

Our review aligns with previous studies, confirming asphyxia as the leading cause of death, followed by trauma and hypothermia. Gaps in knowledge remain on autopsy-confirmed causes of death and related injuries. Standardized forensic protocols could improve death classification accuracy, particularly in cases with combined causes.

## Highlights

• This scoping review is the first focusing on forensic data in avalanche fatalities.
• Only 51 % of postmortem investigations were full internal autopsies.
• Asphyxia was the leading cause of death (72 % of victims with autopsies).
• Combined causes of death (8.5 %) were more frequent than hypothermia alone (1.5 %).
• Trauma-related deaths (18 %) involved fatal head, neck, and thoracic injuries.

## Introduction

Avalanche incidents are responsible for an average of 100 deaths per year in Europe.^1^ Asphyxia is the leading cause of death, followed by trauma and hypothermia.^2^, ^3^, ^4^ The mortality rate exceeds 50 % for critically buried victims (head and chest buried under snow), in contrast to 4 % for partially buried victims.^5^, ^6^ Critical burial may cause airway obstruction with snow, impaired chest expansion caused by the weight and compaction of snow, and hypoxia from rebreathing expired air.^5^ These conditions may all lead to asphyxia. The degree and duration of burial are the main determinants of survival for critically buried victims.^5^ Other factors also influencing the likelihood of survival include airway patency, the presence of an air pocket, snow properties, and the time of day.^5^, ^7^, ^8^

Trauma causes 5–29 % of deaths in avalanche accidents.^9^ The characteristics and severity of associated injuries vary according to the terrain, snow properties, type of outdoor activity, and safety equipment.^2^, ^3^, ^10^ Trauma may impair consciousness reducing the ability to self-extricate or create an air pocket, even in victims with non-life-threatening injuries.^2^, ^3^, ^11^

Hypothermia accounts for only a small proportion of avalanche accident fatalities and generally occurs with critical burial durations of ≥60 min for non-asphyxiated victims.^5^, ^9^ In avalanche incidents, the core temperature of the victim generally decreases by approximately 3 °C/h between the time of the incident and arrival at hospital.^12^ However, extreme cooling rates of 8.5 °C/h, 9 °C/h, 9.4 °C/h and 14 °C/h have been reported.^13^, ^14^, ^15^, ^16^ Progressive cooling can lead to hypothermic cardiac arrest when core temperature drops below 30 °C.^17^ After 90 min, death may also occur from a combination of hypothermia, hypoxia, and hypercapnia, also known as the triple H syndrome.^5^, ^18^

Although some studies of avalanche fatalities report the causes of injury and death, few^2^, ^19^, ^20^ report the results of forensic examinations. The available studies are heterogeneous in terms of study design, setting, and whether postmortem examination was carried out.

We conducted a scoping review to determine the types of studies available and the current understanding of causes of death and injury in forensic investigations conducted on avalanche victims. Our review aimed to summarize existing knowledge and identify knowledge gaps.

## Methods

We conducted a scoping review systematically assessing the available evidence on causes of death and types of injuries of patients who died following avalanche incidents. We specifically searched the literature for information on the causes of death and patterns of injuries based on the findings of the forensic investigations (e.g. postmortem examination, histopathological and imaging findings).

We considered studies including patients of any age and sex who died from avalanche incidents and underwent forensic investigations. We included partially and critically buried victims, excluding patients who were not in outdoor environments (e.g. dead in a building buried by an avalanche). We used the term critical burial to refer to a burial in which the head and chest are buried under snow.^9^ We considered the following studies: epidemiological studies, before and after studies, prospective and retrospective cohort studies, case-control studies, cross-sectional studies, and conference abstracts. We classified retrospective studies with fewer than five victims as case reports or case series. We included evidence synthesis studies (reviews) but excluded them from the analysis. There was no restriction of language or date.

We performed the literature search on July 4, 2024. We designed the literature search with the help of a research librarian. Another information specialist reviewed the final strategies using the Peer Review of Electronic Search Strategies (PRESS) Checklist.^21^ The databases we accessed were PubMed **(**Appendix A**),** Embase.com and Web of Science Core Collection. We searched Google Scholar and the references lists of the included studies on March 18, 2025 for additional papers. We designed the search strategies for Google Scholar in five languages: English, French, German, Spanish, and Italian (Appendix A). We conducted a final search of PubMed on April 22, 2025.

We uploaded the studies identified by the main search to the Zotero program^22^ and eliminated duplicates. One author (CR) screened the titles and abstracts of the retrieved papers to check for eligibility then examined the full texts of collected studies to confirm that they met the inclusion criteria. We applied the same selection process for the final search on PubMed. In case of doubt, another reviewer (MP), blinded to the original results, analyzed the full texts of retrieved studies to validate their inclusion. We used the Rayyan app for systematic reviews during the selection process.^23^ One author (CR) performed the searches on Google Scholar by screening the titles and opening lines of the first 200 results in each language to assess eligibility and analyzed the full texts of the selected articles.

One reviewer (CR) extracted the data from the included studies by using a data extraction table. In case of doubt, a second reviewer (MP) was available to discuss and share data extraction. We resolved disagreements by consensus. We extracted information on the source and design of the studies, on the population (type, sample size, age, sex), and on the type and results of the postmortem examinations (external examination, autopsy, histopathological and imaging findings). We collected information on the cause of death and types of injuries. We coded missing data as “Not reported” and confusing data as “Undetermined”.

The scoping review protocol followed the recommendations of the Scoping Review Protocol Guidance and Scoping Review Protocol Template from the Open Science Framework (OSF) platform.^24^ We registered our protocol on November 17, 2024. The registration is available at the URL: https://osf.io/uhy7j. We followed the Preferred Reporting Items for Systematic Reviews and the Meta-Analyses extension for Scoping Reviews (PRISMA-ScR) statement.^25^ Ethical approval was not required for literature reviews.

## Results

**Fig. 1:**
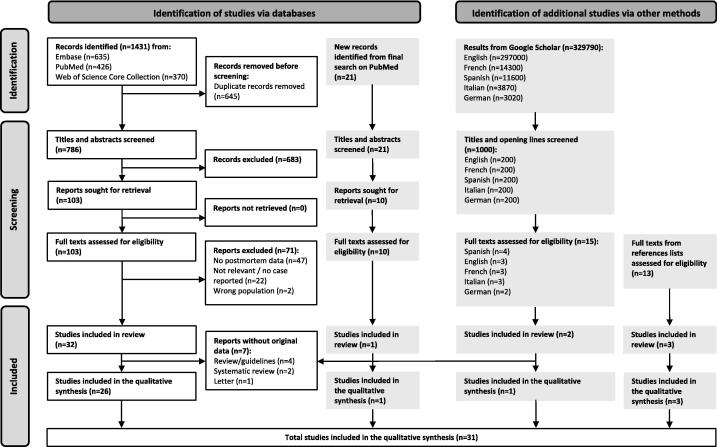
Flow diagram of the scoping review.

Of the 2465 studies we identified, 38 studies met our inclusion criteria. Seven studies did not include original patient data.^20^, ^26^, ^27^, ^28^, ^29^, ^30^, ^31^ The remaining 31 studies were included in the qualitative synthesis (Fig. 1).^11^, ^12^, ^2^, ^3^, ^4^, ^32^, ^33^, ^34^, ^35^, ^36^, ^37^, ^38^, ^39^, ^40^, ^41^, ^42^, ^43^, ^44^, ^45^, ^46^, ^47^, ^48^, ^49^, ^50^, ^51^, ^52^, ^53^, ^54^, ^55^, ^56^, ^57^

All studies were retrospective. Almost half (n = 15, 48 %) came from the USA (n = 9) or Switzerland (n = 6). The median sample size of avalanche-related deaths was 21 (IQR 10–36, range 1–664). Of the 1543 fatalities, 862 (56 %) underwent forensic investigation (Table 1).^11^, ^12^, ^2^, ^3^, ^4^, ^32^, ^33^, ^34^, ^35^, ^36^, ^37^, ^38^, ^39^, ^40^, ^41^, ^42^, ^43^, ^44^, ^45^, ^46^, ^47^, ^48^, ^49^, ^50^, ^51^, ^52^, ^53^, ^54^, ^55^, ^56^, ^57^

**Table 1:** **Summary of the 31 included studies**.^11^, ^12^, ^2^, ^3^, ^4^, ^32^, ^33^, ^34^, ^35^, ^36^, ^37^, ^38^, ^39^, ^40^, ^41^, ^42^, ^43^, ^44^, ^45^, ^46^, ^47^, ^48^, ^49^, ^50^, ^51^, ^52^, ^53^, ^54^, ^55^, ^56^, ^57^ Numbers represent unique patients. PM: postmortem. NR: Not reported. UD: Undetermined.

**First author**	**Year**	**Country**	**Language**	**Study design**	**Avalanche-related deaths**	**Patients with any PM examination**	**Patients without any PM examination**	**Patients with PM examination status not reported**
d’Alnoncourt	2017	France	French	Retrospective	25	25	0	0
Blochliger	1952	Switzerland	German	Retrospective	23	23	0	0
Boyd	2009	Canada	English	Retrospective	204	204	0	0
Christensen	1999	USA	English	Retrospective	17	UD^a^	UD^a^	17
Cohen	2017	France	English	Case reports / case series	5	5	0	0
Daniel	2021	USA	English	Case reports / case series	1	1	0	0
Dorn	1993	Switzerland	German	Retrospective	33	16	NR	17
Eliakis	1974	France	French	Case reports / case series	2	2	0	0
Fieler	2013	Norway	Norwegian	Retrospective	39	5	34	0
Geisenberger	2015	Germany	German	Case reports / case series	2	1	1	0
Gross	2021	Switzerland	English	Retrospective	32	32	0	0
Grosse	2007	Switzerland	English	Case reports / case series	2	1	1	0
Grossman	1989	USA	English	Retrospective	12	1	NR	11
Haegeli	2011	Canada	English	Retrospective	664	143	NR	521
Hohlrieder	2007	Austria	English	Retrospective	36	30	6	0
Johnson	2001	USA	English	Retrospective	28	28	0	0
Kobek	2016	Poland	English	Retrospective	8	6	1	1
Kučerová	2024	Czech Republic	Czech	Retrospective	10	10	0	0
Locher	1996	Switzerland	German	Retrospective	16	2	NR	14
Lugger	1972	Austria	German	Retrospective	20	20	0	0
Mair	1994	Austria	English	Retrospective	11	8	NR	3
Markwalder	1970	Switzerland	German	Retrospective	43	1	NR	42
McIntosh	2007	USA	English	Retrospective	56	56	0	0
McIntosh	2019	USA	English	Retrospective	32	30	2	0
Oshiro	2022	Japan	English	Retrospective	36	26	NR	10
Pigolkin	2012	Russia	Russian	Retrospective	UD^b^	UD^b^	0	UD^b^
Sheets	2018	USA	English	Retrospective	110	110	0	0
Soteras Martínez	2022	Spain	Spanish	Retrospective	42	42	0	0
Stalsberg	1989	Norway	English	Retrospective	18	18	0	0
Tough	1993	USA	English	Retrospective	15	15	0	0
Weston	1977	USA	English	Case reports / case series	1	1	0	0

**Total, n (%)**					**1543 (100 %)**	**862 (56 %)**	**45 (3 %)**	**636 (41 %)**

a The number of avalanche fatalities who underwent PM examination could not be determined, because these cases were reported together with non-avalanche-related deaths. However, PM examination was performed in 30/37 patients.^33^

b The number of avalanche fatalities that underwent PM examination could not be determined, because these cases were reported together with non-avalanche-related deaths. However, PM examination was performed in all patients.^48^

### Postmortem examination

Of the 862 victims who underwent postmortem examination, 442 (51 %) had full internal autopsies while 217 (25 %) had only external examinations. Nine studies did not specify the type of postmortem examination performed, accounting for the remaining 203 victims (24 %).^3^, ^11^, ^33^, ^36^, ^39^, ^42^, ^44^, ^48^, ^54^ Histopathologic findings were available for 48 patients (6 %), postmortem imaging for 12 (1 %), ethanol testing for 189 (22 %) and drug testing for 66 (8 %) (Table 2).^11^, ^12^, ^2^, ^3^, ^4^, ^32^, ^33^, ^34^, ^35^, ^36^, ^37^, ^38^, ^39^, ^40^, ^41^, ^42^, ^43^, ^44^, ^45^, ^46^, ^47^, ^48^, ^49^, ^50^, ^51^, ^52^, ^53^, ^54^, ^55^, ^56^, ^57^

**Table 2:** **Type of the postmortem examinations performed in 862 avalanche fatalities according to the 31 included studies**.^11^, ^12^, ^2^, ^3^, ^4^, ^32^, ^33^, ^34^, ^35^, ^36^, ^37^, ^38^, ^39^, ^40^, ^41^, ^42^, ^43^, ^44^, ^45^, ^46^, ^47^, ^48^, ^49^, ^50^, ^51^, ^52^, ^53^, ^54^, ^55^, ^56^, ^57^ Numbers represent unique patients. PM: postmortem. NR: Not reported. UD: Undetermined.

**First author**	**Year**	**Any PM examination**	**External examination alone**	**Internal autopsy**	**Histopathology**	**Imaging**	**Any toxicology test**	**Ethanol test**	**Drugs test**
d'Alnoncourt	2017	25	0	25	24	7	NR	NR	NR
Blochliger	1952	23	0	23	NR	NR	NR	NR	NR
Boyd	2009	204	87	117	NR	NR	UD^a^	160	55
Christensen	1999	UD^b^	UD^b^	UD^b^	NR	NR	UD^b^	UD^b^	UD^b^
Cohen	2017	5	0	5	5	5	NR	NR	NR
Daniel	2021	1	0	1	NR	NR	NR	NR	NR
Dorn	1993	16	NR	16	NR	NR	NR	NR	NR
Eliakis	1974	2	0	2	NR	NR	NR	NR	NR
Fieler	2013	5	NR	5	NR	NR	NR	NR	NR
Geisenberger	2015	1	0	1	1	NR	1	1	NR
Gross	2021	32	NR	NR	NR	UD^c^	UD^b^	NR	NR
Grosse	2007	1	0	1	NR	NR	NR	NR	NR
Grossman	1989	1	NR	1	NR	NR	NR	NR	NR
Haegeli	2011	143	NR	NR	NR	NR	NR	NR	NR
Hohlrieder	2007	30	NR	30	NR	NR	NR	NR	NR
Johnson	2001	28	NR	NR	NR	NR	NR	NR	NR
Kobek	2016	6	NR	6	6	NR	6	6	6
Kučerová	2024	10	0	10	NR	NR	7	7	4
Locher	1996	2	NR	2	NR	NR	NR	NR	NR
Lugger	1972	20	0	20	UD^d^	NR	NR	NR	NR
Mair	1994	8	NR	8	NR	NR	NR	NR	NR
Markwalder	1970	1	NR	1	NR	NR	NR	NR	NR
McIntosh	2007	56	28	28	NR	NR	UD^e^	UD^e^	UD^e^
McIntosh	2019	30	13	17	NR	NR	NR	NR	NR
Oshiro	2022	26	26	0	NR	NR	NR	NR	NR
Pigolkin	2012	UD^f^	NR	NR	NR	NR	NR	NR	NR
Sheets	2018	110	46	64	NR	NR	NR	NR	NR
Soteras Martínez	2022	42	0	42	NR	NR	NR	NR	NR
Stalsberg	1989	18	6	12	12	NR	NR	NR	NR
Tough	1993	15	11	4	NR	NR	14	14	0
Weston	1977	1	0	1	NR	NR	1	1	1

**Total, n (%)**		**862 (100 %)**	**217 (25 %)**	**442 (51 %)**	**48 (6 %)**	**12 (1 %)**	**UD**	**189 (22 %)**	**66 (8 %)**

a The total number of avalanche fatalities who had toxicological examination was not reported.^2^

b The number of avalanche fatalities who underwent PM examination or had a toxicological examination could not be determined, because these cases were reported together with non-avalanche-related deaths.^33^, ^39^

c The number of avalanche fatalities who underwent PM imaging could not be determined, because these cases were reported together with non-avalanche-related deaths. However, all the cases with autopsy had a PM imaging.^39^

d The number of histological examinations was not reported and was only performed in isolated cases of powder avalanches.^55^

e The number of toxicological examinations performed was not reported. However, one victim tested positive for ethanol and cocaine.^45^

f The number of avalanche fatalities who underwent PM examination could not be determined, because these cases were reported together with non-avalanche-related deaths. However, all victims received a PM examination.^48^

### Causes of death

Among the 1543 fatalities, 693 (45 %) were attributed to asphyxia alone, 164 (11 %) to trauma, 12 (<1%) to hypothermia, and 60 (4 %) to combined causes of death. Other causes of death were reported for 17 victims (1 %).^4^, ^44^, ^50^, ^56^ Causes of death were not reported in 597 cases (39 %). The causes of death were determined either through internal autopsy, external examination conducted by coroners or forensic physicians, or by physicians in the emergency room or in pre-hospital settings. In most cases, the type of examination performed or the qualifications of the personnel involved in determining the cause of death were not reported.

Of the 442 avalanche victims who underwent internal autopsy, the cause of death was reported in 387 cases. Asphyxia was identified in 279 (72 %), trauma in 69 (18 %), hypothermia in 6 (2 %) and combined causes of death in 33 (9 %) (Table 3).^12^, ^32^, ^40^, ^41^, ^2^, ^3^, ^4^, ^35^, ^36^, ^37^, ^38^, ^43^, ^44^, ^45^, ^49^, ^50^, ^51^, ^52^, ^53^, ^54^, ^55^, ^56^, ^57^

**Table 3:** **Causes of death of the 387 avalanche fatalities who underwent internal autopsy and for whom the cause of death was reported**.^12^, ^32^, ^40^, ^41^, ^2^, ^3^, ^4^, ^35^, ^36^, ^37^, ^38^, ^43^, ^44^, ^45^, ^49^, ^50^, ^51^, ^52^, ^53^, ^54^, ^55^, ^56^, ^57^ Results are reported as n (%).

**First author**	**Year**	**Asphyxia**	**Trauma**	**Hypothermia**	**Combined** **Asphyxia and Trauma**	**Combined** **Asphyxia and Hypothermia**	**Combined** **Trauma and Hypothermia**	**Combined** **Asphyxia, Trauma and** **Hypothermia**
d'Alnoncourt	2017	18	1		6			
Blochliger	1952	20	1			2		
Boyd	2009	80	25		12			
Daniel	2021			1				
Dorn	1993	15	1					
Eliakis	1974	1						
Fieler	2013	4	1					
Geisenberger	2015					1		
Grosse	2007					1		
Grossman	1989		1					
Hohlrieder	2007		2	1				
Kobek	2016	6						
Kučerová	2024	3	1		1	2	2	1
Locher	1996	2						
Lugger	1972	19	1					
Mair	1994	6	2					
Markwalder	1970	1						
McIntosh	2007	23	2		3			
Sheets	2018	42	17					
Soteras Martínez	2022	24	14	4				
Stalsberg	1989	10			2			
Tough	1993	4						
Weston	1977	1						

**Total, n (%)**		**279 (72 %)**	**69 (18 %)**	**6 (2 %)**	**24 (6 %)**	**6 (2 %)**	**2 (0.5 %)**	**1 (0.3 %)**

### Autopsy findings associated with deaths by asphyxia

Eight studies reported detailed autopsy findings for 63 patients whose death was attributed to asphyxia alone (Appendix B).^32^, ^43^, ^56^, ^51^, ^52^, ^53^ The most frequently documented findings were liquid blood in the organs or vessels, attributed to asphyxia in 39 cases (62 %), acute organ congestion in 37 (59 %), dilation of the right ventricle in 24 (38 %), pulmonary edema or congestion in 22 (35 %), severe general cyanosis in 20 (32 %), and cerebral edema in 19 (30 %). Petechial hemorrhages, typically considered as markers of hypoxia, were found in multiple organs, including the pleura in 13 patients (21 %), stomach in 11 (17 %), pericardium in 10 (16 %), brain in 6 (10 %), conjunctivae in 6 (10 %), skin in 3 (5 %), and unreported sites in 4 (6 %). The most frequently reported signs of trauma included minor skin or musculoskeletal injuries. Injuries involving the head, thorax, and pelvis appeared more severe and may have contributed to death, although the original studies did not report trauma as a cause or contributor of death. The only finding attributed to cold exposure was frostbite in two victims.

### Autopsy findings associated with deaths caused by trauma

Trauma was the second most common cause of death among the 387 victims who underwent full internal autopsies for whom the cause of death was reported, accounting for 69/387 cases (18 %). Seven studies reported detailed autopsy findings for nine trauma-related deaths (Appendix C).^3^, ^32^, ^41^, ^45^, ^53^, ^55^, ^57^ Most injuries involved the head and neck (n = 8, 90 %), and chest (n = 4, 44 %). Fatal injuries reported included two cases of isolated cervical fracture with dislocation, one case of atlanto-occipital dislocation, one case of aortic transection, and one case of unspecified fatal traumatic brain injury. The fatal injuries were not reported for the remaining four cases.

### Autopsy findings associated with hypothermia deaths

Hypothermia was the least frequent single cause of death reported among the 387 victims who underwent full internal autopsies and for whom the cause of death was reported, accounting for 2 % (6/387) of cases. No autopsy findings indicating hypothermia were reported except for Wischnewski spots in a single case from one study.^3^

### Autopsy findings associated with combined causes of death

The most frequent combined cause of death among the 387 victims who underwent internal autopsy, for whom the cause of death was reported, was asphyxia associated with trauma (n = 24, 6 %), followed by asphyxia and hypothermia (n = 6, 2 %), trauma and hypothermia (n = 2, 0.5 %) and asphyxia and trauma/hypothermia (n = 1, 0.3 %). We present detailed autopsy findings of the nine combined asphyxia and trauma-related deaths (Appendix D).^50^, ^53^, ^57^ We also present the detailed autopsy findings of the five combined asphyxia-hypothermia-related deaths (Appendix E**)**.^32^, ^38^, ^57^ Signs of cold exposure or general hypothermia included Wischnewski spots (n = 3), frostbite (n = 1) and cloudy corneas (n = 1).^32^, ^38^, ^57^

### Histopathological examination

Five studies reported the results of histopathological examinations in 48 patients (Appendix F**)**.^34^, ^38^, ^43^, ^50^, ^53^ The cases from another study^55^ were not included due to insufficient data. Most findings were related to asphyxia. Pulmonary pathology was observed in all victims. Frequent findings included serosanguinous edema, intra-alveolar hemorrhages, and vascular congestion. Histopathological findings suggestive of hypothermia were infrequently reported. These included vacuolar loss in the pancreas (n = 11) and fatty degeneration of kidney tubules (n = 9) or myocardial cells (n = 2).

### Imaging

Of the 31 included studies, three^34^, ^39^, ^53^ reported the use of postmortem imaging. One study did not report the number of avalanche fatalities that underwent postmortem imaging because these cases were reported together with non-avalanche-related deaths.^39^ Radiological findings were available for 2 studies with a total of 12 cases.^34^, ^53^ The findings were similar in both studies, described as consistent with cardiogenic or negative-pressure pulmonary edema (Table 4).^34^, ^53^

**Table 4:** **Radiological findings for the 12 avalanche fatalities with postmortem imaging**.^34^, ^53^ Results are reported as n (%). NR: Not reported.

	**d’Alnoncourt, 2017** **n = 7**	**Cohen, 2017** **n = 5**	**Total** **n = 12**
Ground-glass opacities	5 (71 %)	5 (100 %)	10 (83 %)
Peribronchovascular thickening	5 (71 %)	5 (100 %)	10 (83 %)
Consolidations or focal consolidations	3 (43 %)	2 (40 %)	5 (42 %)
Septal interlobular thickening	2 (29 %)	2 (40 %)	4 (33 %)
Micronodules, centrilobular micronodules or micronodular infiltration	2 (29 %)	3 (60 %)	5 (42 %)
Bronchial or distal bronchial lesions	6 (86 %)	NR	6 (50 %)
Tracheal lesions	2 (29 %)	NR	2 (17 %)

### Toxicologic examination

Nine studies reported postmortem toxicological examinations.^2^, ^33^, ^38^, ^39^, ^43^, ^45^, ^51^, ^52^, ^57^ One study reported 160 ethanol and 55 cannabinoid tests.^2^ Most ethanol tests were performed on blood samples (n = 167),^2^, ^57^ followed by combined analyses of blood, urine, and vitreous humor (n = 14),^51^ and combined blood and urine testing (n = 1).^38^ In six cases, because of advanced decomposition of the bodies, ethanol analysis was conducted using pleural cavity fluid and tissue samples from the liver and kidney.^43^ Of the 189 victims tested for ethanol, seven (4 %) were positive.^2^, ^45^, ^51^ Among the 66 victims tested for drugs, cannabinoids were detected in seven (11 %) victims, and cocaine in one victim (1 %).^2^, ^45^

## Discussion

As far as we know, ours is the first study to report forensic information on avalanche victims systematically. Most studies on avalanche-related fatalities have relied on presumed diagnoses provided by the rescuers. Significant uncertainty remains in prehospital settings, where clinical examination has only moderate accuracy in detecting injuries, and access to diagnostic tools is limited.^58^, ^59^, ^60^, ^61^ The present study may provide greater insight into determining the cause of death in avalanche victims.

### Causes of death in avalanche fatalities

Among the 1543 fatalities, the type of examination performed and the qualifications of the personnel involved in determining the cause of death were heterogenous or not reported. To ensure data interpretability and comparability, only the 387 autopsy-confirmed causes of death were presented. Asphyxia accounted for 72 % of deaths among the avalanche victims who underwent autopsy, followed by trauma (18 %), and hypothermia (2 %). Our findings are consistent with prior studies. These also reported asphyxia as the most common cause of death (65–100 %), followed by trauma (5–29 %), and hypothermia (0–4 %).^9^ We identified combined causes of death in 9 % of cases, a finding not consistently reported in previous reviews. This highlights that numerous factors can contribute to avalanche fatalities and may indicate a need for more extensive, systematic, postmortem investigations to determine the causes of death in avalanche victims.^20^, ^62^

### Autopsy findings in avalanche fatalities

Asphyxia-related findings were the most frequent findings documented at autopsy. These included signs of hypoxia, such as central cyanosis, pulmonary findings, such as alveolar edema; and circulatory disturbances secondary to asphyxia, including visceral congestion and petechial hemorrhages. Avalanche victims often have prolonged hypoxia associated with hypercapnia, except in cases of complete airway obstruction. Autopsy findings were often obvious and pronounced with prolonged asphyxia in contrast to sudden death by asphyxia in which asphyxial features may be minimal or absent.^63^ Traumatic injuries mainly involved the head and neck, followed by the thorax. This supports recommendations to provide spinal motion restriction when indicated during the management of avalanche victims.^9^

Abdominal or pelvic injuries were infrequent. Autopsy findings associated with hypothermia or cold exposure were also uncommon, likely because asphyxia was the main cause of death. Few victims died from accidental hypothermia.^9^ The postmortem definite diagnosis of hypothermia is often difficult and is frequently made by excluding other causes of death in combination with circumstantial evidence of cold exposure.^64^ However, hypothermia is often associated with frostbite and Wischnewski spots. Pulmonary edema may also occur in hypothermia, but diagnostic value was limited unless pulmonary edema was associated with other signs of hypothermia.^63^

### Traumatic injuries in avalanche fatalities

Trauma is frequently reported in avalanche fatalities, particularly in cases involving significant injuries. In critically buried avalanche victims in cardiac arrest, lethal injuries, such as decapitation or truncal transection, are contraindications to cardiopulmonary resuscitation (CPR).^9^, ^28^, ^58^, ^65^, ^66^ Although this recommendation may appear self-evident, injuries described in the studies were often not immediately recognizable as fatal. Decisions to terminate resuscitation because of fatal injuries made in a prehospital setting are based on clinical assessment, in which sensitivity is limited. On the other hand, trauma may initially be suspected as the cause of death but ruled out at autopsy.

Some traumatic injuries were described as having been caused by CPR,^67^, ^68^, ^69^ including aspiration of gastric contents, intrathoracic bleeding (pericardial or pleural), rib fractures, and subsequent complications such as fat embolism or pneumothorax. Of the 37 victims who received CPR for whom autopsy results and causes of death were reported, only two sustained injuries attributable to resuscitation.^32^, ^38^, ^50^, ^53^, ^56^ The first victim had aspiration of gastric contents following vomiting during resuscitation as well as a pneumothorax and a fat embolism caused by rib fractures.^56^ These injuries were attributed to CPR based on their location posterior to the axillary line. In the second victim, aspiration of gastric content was also possibly related to resuscitation.^50^ We were unable to document autopsy findings for cases in which the causes of death were not reported (Appendices B-E). One study reported that 12 of 16 rib or sternal fractures identified at autopsy were attributed to CPR.^3^ In another study, there were nine documented cases of fat embolism potentially related to resuscitation, although no rib fractures were attributed to resuscitation.^55^ Six cases of massive aspiration following vomiting were also reported and possibly resulting from resuscitation attempts.^55^ Our findings support current recommendations against performing thoracostomies in avalanche victims in cardiac arrest without suspected hemothorax. Such procedures may cause bleeding and complicate rewarming with extracorporeal life support (ECLS), if indicated.^9^, ^70^, ^71^ Ultrasound may be used to assess the presence of pneumothorax or hemothorax in avalanche victims.

### Postmortem investigations of avalanche fatalities

Postmortem investigations varied considerably among studies, reflecting heterogeneous techniques used at various medical centers. Only three studies mentioned the use of postmortem imaging.^34^, ^39^, ^53^ In one study, a noncontrast postmortem cervico thoraco abdominopelvic computed tomography (CT) scan (“panscan”) was performed on seven victims, but only thoracic findings were reported.^53^ In six of the seven cases pulmonary edema or vascular congestion was seen on postmortem imaging. In a study that exclusively used postmortem chest CT, all five cases had similar abnormalities such as ground-glass opacities, peribronchovascular and septal interlobular thickening, consolidations, and micronodules.^34^ The limited use of postmortem imaging may have been a result of the recent development of this technique, the significant costs involved, and restricted access to imaging equipment and resources. Discrepancies between the radiological and histopathological findings highlight the potential role of imaging in complementing autopsies and mitigating sampling bias.^53^

Histopathological examination was reported in six studies.^34^, ^38^, ^43^, ^50^, ^53^, ^55^ Hematoxylin-eosin (H&E) staining was the most frequent technique, used in three studies.^34^, ^43^, ^53^ Myocardial staining with Luxol Fast Blue was used in one study. This showed myocardial contraction band necrosis in six cases, consistent with extreme ischemic ventricular contracture caused by hypoxia and circulatory disturbances.^50^ One study presented a detailed methodological description, with the use of Movat's stain to assess pulmonary emphysema, and a “special” red oil stain that was applied to all organ slides to assess for fatty degeneration.^53^ Two of the six studies did not mention the techniques used for histopathological examination.^38^, ^55^ In one study, specific staining techniques were required because of the advanced state of body decomposition. The authors used Gomori staining to detect acute pulmonary emphysema and AZAN staining to detect the erythrocytes in intra-alveolar and interstitial hemorrhages.^43^ In another study, the brain, including the cerebellum, was examined in only four cases, while other organs were assessed in all 24 cases.^53^ Fatty degeneration was evaluated in 18 kidney and heart samples, and in 17 pancreatic samples.^53^ One of the six studies^34^ focused exclusively on the examination of the lungs.

For toxicological examination, one study^51^ reported ethanol concentrations of 20 mg/dL in blood and vitreous humor and 10 mg/dL in urine. Another study^2^ used a higher threshold, considering blood ethanol levels ≥ 80 mg/dL as positive.

The knowledge resulting from this review provides greater awareness of the main causes of death in avalanche victims and helps to clarify priorities for rescue and initial treatment. The predominance of asphyxia highlights the importance of rapid extrication and airway management, as described in current practice recommendations.^9^ The great proportion of trauma-related deaths underlines the need for early recognition and management of severe injuries in the prehospital setting. Although hypothermia was less frequently identified as the sole cause of death for avalanche victims, its presence is associated with higher chances of survival, which underscores the importance of logistic coordination and timely transfer to ECLS centers. While the translation of post-mortem findings into patient management is challenging, these conclusions support current recommendations and contribute to refining priorities for avalanche rescue.

## Limitations

The retrospective design limited the overall level of evidence. Heterogeneity among studies and potential selection bias reduced our ability to draw definitive conclusions from our results. Postmortem examinations were conducted by various professionals, including coroners and forensic physicians. This may have contributed to variability in findings and reporting. Histopathological methods varied across studies. Postmortem imaging was rarely performed. The term “autopsy” was not clearly defined in some of the studies. Some autopsies may have involved external examinations only, introducing a potential bias. We classified cases as internal autopsies when the original studies explicitly mentioned “autopsy” or when the findings could not have been observed by external examination alone.

For 11 victims with autopsy data included in the qualitative synthesis, details about outdoor exposure were unclear or undetermined.^32^ Among the victims who were not autopsied or did not have a postmortem examination, eight were found in houses or vehicles.^36^, ^56^ One young soldier voluntarily buried himself during a training exercise.^55^ Because these cases with outdoor victims were reported collectively, they could not be excluded from the dataset. They may have influenced the reported proportions of causes of death and autopsy findings.

Selection bias cannot be excluded because the design and methods of some studies may have influenced the conclusions about causes of death. Two studies included only hypothermic patients with cardiac arrest.^12^, ^44^ Another explicitly classified causes of death as being either asphyxia or blunt trauma.^11^ In one study, asphyxia was considered a diagnosis of exclusion when no fatal injury was identified on examination. This increases the possibility of overdiagnosis.^46^ Some deaths related to trauma or hypothermia may have been missed, because not all cases underwent a full internal autopsy.

## Conclusion

We reviewed studies of the forensic aspects of avalanche fatalities and integrated current knowledge about the causes of death and injuries of victims. Our results aligned with previous studies, confirming asphyxia as the leading cause of death in avalanches, followed by trauma and hypothermia. We also identified combined causes of death that were not systematically reported in previous literature, highlighting the potential value of forensic investigations in accurate determination of the mechanisms of death. We identified knowledge gaps, including the limited data on autopsy-confirmed causes of death, and findings of postmortem investigations. Standardized forensic protocols integrating autopsy, histopathology, toxicology, and imaging might enhance our understanding of injury patterns and improve the classification of avalanche-related deaths. Our results may be relevant for researchers, clinicians, or other professionals concerned with avalanche fatalities.

## Support and funding

None.

## CRediT authorship contribution statement

**Céliane Romy:** Writing – original draft, Visualization, Investigation, Conceptualization. **David Eidenbenz:** Writing – review & editing, Visualization, Conceptualization. **Silke Grabherr:** Writing – review & editing, Validation, Conceptualization. **Ken Zafren:** Writing – review & editing, Conceptualization. **Cécile Jaques:** Writing – review & editing, Methodology, Conceptualization. **Nicolas Hall:** Writing – review & editing, Conceptualization. **Mathieu Pasquier:** Writing – review & editing, Visualization, Supervision, Methodology, Conceptualization.

## Declaration of competing interest

The authors declare that they have no known competing financial interests or personal relationships that could have appeared to influence the work reported in this paper.
