# Supplementary material for: Causes of death and types of injuries of avalanche fatalities based on forensic data: a scoping review
Source: Resusc Plus. 2025 Sep 13;26:101101. doi: 10.1016/j.resplu.2025.101101 (PMC12506530; doi:10.1016/j.resplu.2025.101101)
Supplement: Supplementary Data 1 [file mmc1.pdf]

Appendix A. Complete strategies for the literature search.

Complete strategy for PubMed.

("Avalanches"[Mesh] OR (Avalanche*[tiab] AND ("snow"[MeSH] OR "Ice"[Mesh:NoExp] OR "Snow Sports"[Mesh] OR "Skiing"[Mesh] OR "Mountaineering"[Mesh] OR "Wilderness Medicine"[Mesh] OR "Wilderness"[Mesh] OR "Disaster Medicine"[Mesh] OR "Natural Disasters"[Mesh:noexp] OR "Disasters"[Mesh:noexp] OR "Mass Casualty Incidents"[Mesh:noexp] OR "Accidents"[Mesh:NoExp] OR "Himalayas"[Mesh] "European Alpine Region"[Mesh] OR "Altitude"[Mesh] OR "Disaster Victims"[Mesh] OR "Survivors"[Mesh:NoExp] OR "Rescue Work"[Mesh] OR "Emergency Medical Services"[Mesh:noexp] OR "Emergencies"[Mesh] OR "Life Support Care"[Mesh:noexp] OR "Emergency Treatment"[Mesh] OR "Air Ambulances"[Mesh] OR "Rewarming"[Mesh] OR "Cold Injury"[Mesh] OR "Wounds and Injuries"[Mesh] OR "Injury Severity Score"[Mesh] OR "Abbreviated Injury Scale"[Mesh] OR "Hypothermia"[Mesh] OR "Heart Arrest"[Mesh] OR "Vital Signs"[Mesh:NoExp] OR "Survival Rate"[Mesh] OR "Survival"[Mesh] OR "Body Temperature"[Mesh] OR (snow*[tiab] OR ice[tiab] OR "winter sport*"[tiab] OR Skiing[tiab] OR Skier*[tiab] OR Freeski*[tiab] OR Freerid*[tiab] OR Backcountry[tiab] OR "Off-piste"[tiab] OR "Off piste"[tiab] OR Offpiste[tiab] OR sledding[tiab] OR hike*[tiab] OR hiking[tiab] OR trek*[tiab] OR climb*[tiab] OR mount*[tiab] OR outdoor*[tiab] OR wilderness[tiab] OR disaster*[tiab] OR "mass casualty incident*"[tiab] OR Accident*[tiab] OR Alps[tiab] OR Alpin*[tiab] OR Andes[tiab] OR Himalaya*[tiab] OR "Rocky mountain*"[tiab] OR "Atlas mountain*"[tiab] OR Pyrene*[tiab] OR Highland*[tiab] OR Altitude[tiab] OR Victim*[tiab] OR surviv*[tiab] OR Nonsurviv*[tiab] OR "non-surviv*"[tiab] OR "non surviv*"[tiab] OR Trauma*[tiab] OR Buried[tiab] OR burial[tiab] OR Emergenc*[tiab] OR Helicopter*[tiab] OR "air ambulance*"[tiab] OR "aeromedical transport*"[tiab] OR "aero medical transport"[tiab:~0] OR "aero medical transports"[tiab:~0] OR "aero medical transportation"[tiab:~0] OR "air medical transport*"[tiab] OR Rescu*[tiab] OR resuscitat*[tiab] OR reanimat*[tiab] OR "first aid"[tiab] OR "life support*"[tiab] OR rewarm*[tiab] OR warm*[tiab] OR injur*[tiab] OR wound*[tiab] OR Hypotherm*[tiab] OR "heart arrest*"[tiab] OR "cardiac arrest*"[tiab] OR asystol*[tiab] OR Asphyxi*[tiab] OR suffocat*[tiab] OR "airway patency"[tiab] OR "air pocket*"[tiab] OR Extricat*[tiab] OR "core temperature"[tiab:~3] OR "body temperature"[tiab:~3] OR "vital sign*"[tiab] OR frostbite*[tiab] OR "frost bite*"[tiab] OR "frost-bite*"[tiab])))) AND ("Death"[Mesh] OR "Wounds and Injuries"[Mesh] OR "Disaster Victims"[Mesh] OR "Autopsy"[Mesh] OR "Cause of Death"[Mesh] OR "Mortality"[Mesh:noexp] OR "Mortality"[subheading] OR "Fatal Outcome"[Mesh] OR "Forensic Sciences"[Mesh] OR "Diagnostic Imaging"[Mesh] OR "diagnostic imaging"[subheading] OR "Coroners and Medical Examiners"[Mesh] OR "Pathology"[Mesh] OR "pathology"[Subheading] OR "physiopathology"[Subheading] OR (injur*[tiab] OR trauma*[tiab] OR victim*[tiab] OR Nonsurviv*[tiab] OR "non-surviv*"[tiab] OR "non surviv*"[tiab] OR Death*[tiab] OR Dead*[tiab] OR Died[tiab] OR Decease*[tiab] OR Killed[tiab] OR killing[tiab] OR Fatal*[tiab] OR Lethal*[tiab] OR mortality[tiab] OR Postmortem[tiab] OR "post-mortem"[tiab] OR "post mortem"[tiab] OR Cadaver*[tiab] OR corpse*[tiab] OR Autops*[tiab] OR Necrops*[tiab] OR Obduction*[tiab] OR Forensic*[tiab] OR Medicolegal[tiab] OR "medico-legal"[tiab] OR "medico legal"[tiab] OR "legal medicine"[tiab] OR Coroner*[tiab] OR "Medical examiner*"[tiab] OR "legal examination"[tiab:~3] OR "legal exam"[tiab:~3] OR "corpse examination"[tiab:~3] OR "corpse exam"[tiab:~3] OR "legal exams"[tiab:~3] OR "corpse exams"[tiab:~3] OR Physiopatholog*[tiab] OR Patholog*[tiab] OR Histopatholog*[tiab] OR Imaging[tiab] OR Tomograph*[tiab] OR CT[tiab] OR Scan[tiab] OR scanner[tiab] OR "Magnetic resonance"[tiab] OR MRI[tiab] OR MR[tiab] OR radiolog*[tiab] OR radiograph*[tiab] OR roentgenograph*[tiab] OR radioimaging[tiab] OR "radio-imaging"[tiab] OR "X-ray"[tiab] OR "X-rays"[tiab] OR "X ray"[tiab] OR "X rays"[tiab] OR "gamma-ray"[tiab] OR "gamma-rays"[tiab] OR "gamma ray"[tiab] OR "gamma rays"[tiab] OR PMCT[tiab] OR PMMR[tiab] OR "cause injury"[tiab:~3] OR "causes injury"[tiab:~3] OR "causes injuries"[tiab:~3] OR "cause trauma"[tiab:~3] OR "causes trauma"[tiab:~3]))

Complete strategy for Embase.com.

(('avalanche'/de OR Avalanche*:ab,ti,kw) AND ('snow'/de OR 'ice'/de OR 'winter sport'/de OR 'skiing'/de OR 'skier'/de OR 'snowmobile'/de OR 'sledding'/de OR 'hiking'/de OR 'hiker'/de OR 'trekking'/de OR 'trekker'/de OR 'climbing sport'/exp OR 'outdoor'/de OR 'outdoor activity'/de OR 'wilderness medicine'/de OR 'wilderness'/de OR 'disaster medicine'/de OR 'natural disaster'/de OR 'disaster'/de OR 'mass disaster'/de OR 'accident'/de OR 'mountain ranges'/exp OR 'alpine'/de OR 'mountain'/de OR 'altitude'/de OR 'victim'/de OR 'disaster victim'/de OR 'survivor'/de OR 'non survivor'/de OR 'rescue work'/de OR 'emergency health service'/de OR 'emergency'/de OR 'emergency treatment'/exp OR 'emergency evacuation'/de OR 'air medical transport'/exp OR 'helicopter'/de OR 'warming'/de OR 'cold injury'/exp OR 'injury'/exp OR 'heart arrest'/exp OR 'asphyxia'/de OR 'suffocation'/de OR 'airway patency'/de OR 'vital sign'/de OR 'survival'/exp OR 'core temperature'/de OR 'body temperature'/exp OR (snow* OR ice OR "winter sport*" OR Skiing OR Skier* OR Freeski* OR Freerid* OR Backcountry OR "Off-piste" OR "Off piste" OR Offpiste OR sledding OR hike* OR hiking OR trek* OR climb* OR mount* OR outdoor* OR wilderness OR disaster* OR "mass casualty incident*" OR Accident* OR Alps OR Alpin* OR Andes OR Himalaya* OR "Rocky mountain*" OR "Atlas mountain*" OR Pyrene* OR Highland* OR Altitude OR Victim* OR surviv* OR Nonsurviv* OR "non-surviv*" OR "non surviv*" OR Trauma* OR Buried OR burial OR Emergenc* OR Helicopter* OR "air ambulance*" OR "aeromedical transport*" OR "aero-medical transport*" OR "air medical transport*" OR Rescu* OR resuscitat* OR reanimat* OR "first aid" OR "life support*" OR rewarm* OR warm* OR injur* OR wound* OR Hypotherm* OR "heart arrest*" OR "cardiac arrest*" OR asystol* OR Asphyxi* OR suffocat* OR "airway patency" OR "air pocket*" OR Extricat* OR ((core or body) NEXT/3 temperature) OR "vital sign*" OR frostbite* OR "frost bite*" OR "frost-bite*"):ab,ti,kw)) AND ('injury'/exp OR 'victim'/de OR 'disaster victim'/de OR 'non survivor'/de OR 'death'/exp OR 'autopsy'/exp OR 'cause of death'/exp OR 'mortality'/de OR 'mortality rate'/exp OR 'killing'/de OR 'forensic science'/exp OR 'medicolegal aspect'/de OR 'diagnostic imaging'/exp OR 'coroner'/de OR 'pathology'/de OR 'pathophysiology'/de OR 'histopathology'/de OR (injur* OR trauma* OR victim* OR Nonsurviv* OR "non-surviv*" OR "non surviv*" OR Death* OR Dead* OR Died OR Decease* OR Killed OR killing OR Fatal* OR Lethal* OR mortality OR Postmortem OR "post-mortem" OR "post mortem" OR Cadaver* OR corpse* OR Autops* OR Necrops* OR Obduction* OR Forensic* OR Medicolegal OR "medico-legal" OR "medico legal" OR "legal medicine" OR Coroner* OR "Medical examiner*" OR ((legal OR corpse) NEAR/3 exam*) OR Physiopatholog* OR Patholog* OR Histopatholog* OR Imaging OR Tomograph* OR CT OR Scan OR scanner OR "Magnetic resonance" OR MRI OR MR OR radiolog* OR radiograph* OR roentgenograph* OR radioimaging OR "radio-imaging" OR "X-ray" OR "X-rays" OR "X ray" OR "X rays" OR "gamma-ray" OR "gamma-rays" OR "gamma ray" OR "gamma rays" OR PMCT OR PMMR OR (caus* NEAR/3 (injur* OR trauma*))):ab,ti,kw)

Complete strategy for Web of Science Core Collection. Option: Exact Search.

((TS=(Avalanche* AND (snow* OR ice OR "winter sport*" OR skiing OR skier* OR freeski* OR Freerid* OR Backcountry OR "Off-piste" OR "Off piste" OR offsite OR sledding OR hiking OR hike* OR trek* OR climb* OR mount* OR outdoor* OR wilderness OR disaster* OR accident* OR "mass casualty incident*" OR victim* OR surviv* OR "non surviv*" OR Nonsurviv* OR "non-surviv*" OR rescu* OR emergenc* OR "air medical transport*" OR "air ambulance*" OR "aeromedical transport*" OR "aero-medical transport*" OR helicopter* OR warm* OR rewarm* OR injur* OR wound* OR "heart arrest*" OR "cardiac arrest*" OR asystol* OR asphyxi* OR suffocat* OR "airway patency" OR "air pocket*" OR "vital sign*" OR ((core OR body) NEAR/3 temperature) OR Hypotherm* OR altitude OR alpin* OR Alps OR Andes OR Himalaya* OR "Rocky mountain*" OR "Atlas mountain*" OR Pyrene* OR Highland* OR Trauma* OR Buried OR burial OR resuscitat* OR reanimat* OR "first aid" OR "life support*" OR Extricat* OR frostbite* OR "frost bite*" OR "frost-bite*"))) AND (TS=(injur* OR trauma* OR victim* OR "non surviv*" OR nonsurviv* OR "non-surviv*" OR death* OR Dead* OR Died OR Decease* OR Cadaver* OR corpse* OR autops* OR Necrops* OR Obduction* OR Fatal* OR Lethal* OR mortality OR killing OR killed OR forensic* OR Postmortem OR "post-mortem" OR "post mortem" OR medicolegal OR "medico-legal" OR "medico legal" OR "legal medicine" OR Coroner* OR "Medical examiner*" OR ((legal OR corpse) NEAR/3 exam*) OR Physiopatholog* OR Patholog* OR Histopatholog* OR Imaging OR Tomograph* OR CT OR Scan OR scanner OR "Magnetic resonance" OR MRI OR MR OR radiolog* OR radiograph* OR roentgenograph* OR radioimaging OR "radio-imaging" OR "X-ray" OR "X-rays" OR "X ray" OR "X rays" OR "gamma-ray" OR "gamma-rays" OR "gamma ray" OR "gamma rays" OR PMCT OR PMMR OR (caus* NEAR/3 (injur* OR trauma*))))) AND (SU=("PUBLIC ENVIRONMENTAL OCCUPATIONAL HEALTH" OR "SPORT SCIENCES" OR "GENERAL INTERNAL MEDICINE" OR "EMERGENCY MEDICINE" OR "RADIOLOGY NUCLEAR MEDICINE MEDICAL IMAGING" OR "SURGERY" OR "NEUROSCIENCES NEUROLOGY" OR "PSYCHIATRY" OR "BIOCHEMISTRY MOLECULAR BIOLOGY" OR "PSYCHOLOGY" OR "CARDIOVASCULAR SYSTEM CARDIOLOGY" OR "LIFE SCIENCES BIOMEDICINE OTHER TOPICS" OR "OPERATIONS RESEARCH MANAGEMENT SCIENCE" OR "LEGAL MEDICINE" OR "PHYSIOLOGY" OR "ROBOTICS" OR "ANESTHESIOLOGY" OR "ORTHOPEDICS" OR "ONCOLOGY" OR "RESPIRATORY SYSTEM" OR "DERMATOLOGY" OR "PATHOLOGY" OR "TRANSPORTATION" OR "CELL BIOLOGY" OR "FOOD SCIENCE TECHNOLOGY" OR "NURSING" OR "OPHTHALMOLOGY" OR "OTORHINOLARYNGOLOGY" OR "PHARMACOLOGY PHARMACY" OR "RESEARCH EXPERIMENTAL MEDICINE" OR "HEALTH CARE SCIENCES SERVICES" OR "IMMUNOLOGY" OR "PEDIATRICS" OR "SPECTROSCOPY" OR "TRANSPLANTATION" OR "ANATOMY MORPHOLOGY" OR "BIOMEDICAL SOCIAL SCIENCES" OR "BIOTECHNOLOGY APPLIED MICROBIOLOGY" OR "DENTISTRY ORAL SURGERY MEDICINE" OR "DEVELOPMENT STUDIES" OR "ENTOMOLOGY" OR "GENETICS HEREDITY" OR "GERIATRICS GERONTOLOGY" OR "HEMATOLOGY" OR "INFECTIOUS DISEASES" OR "MEDICAL INFORMATICS" OR "MICROSCOPY" OR "PHILOSOPHY" OR "VIROLOGY"))

Complete strategies for Google Scholar.

English:

avalanche autopsy|forensic|imaging|radiology|tomography|medicolegal|postmortem|"cadaver|corpse examination"|pathology|histopathology|"legal medicine"|coroners|"medical examiners"|"cause|causes|patterns|pattern AROUND(3) death|injury|injuries"

French:

avalanche autopsie|forensique|imagerie|radiologie|tomographie|médico-légal|postmortem|"examen|expertise cadavre|corps"|pathologie|histopathologie|"médecine légale"|légiste|"cause|causes|mécanisme|mécanismes AROUND(3) mort|blessure|blessures|lésion|lésions"

German:

Autopsie|Forensik|Bildgebende|Radiologie|Tomografie|rechtsmedizinisch|gerichtsmedizinisch|postmortem|"Untersuchung Körper|Leiche"|Pathologie|Histopathologie|Rechtsmedizin|Gerichtsmediziner|Todesursache|"Ursache|Mechanismus AROUND(3) Tod|Verletzungen"

Spanish:

avalancha autopsia|forense|imagen|radiología|tomografía|medico-legale|postmortem|"examen cadáver|cuerpo"|patología|histopatología|"medicina legal"|"causa|causas|mecanismo|mecanismos AROUND(3) muerte|lesión|lesiones"

Italian:

valanga autopsia|forense|radiologia|tomografia|"medico legale"|postmortem|"esame cadavere|corpo"|patologia|istopatologia|"medicina legale"|"causa|cause|meccanismo|meccanismi AROUND(3) morte|lesione|lesioni|ferita|feriti"
