# Supplementary material for: Causes of death and types of injuries of avalanche fatalities based on forensic data: a scoping review
Source: Resusc Plus. 2025 Sep 13;26:101101. doi: 10.1016/j.resplu.2025.101101 (PMC12506530; doi:10.1016/j.resplu.2025.101101)
Supplement: Supplementary Data 2 [file mmc2.pdf]

Appendix B. Autopsy findings for the 63 avalanche victims whose death was attributed to asphyxia alone [32,43,50–53,56,57]. Lesions/injuries attributed to trauma or hypothermia were not reported as being or contributing to the cause of death on the original studies.

Findings by anatomical regions (number of victims)	Lesions/injuries associated with asphyxia (n)	Lesions/injuries attributed to trauma (n)	Lesions/injuries attributed to cold or hypothermia (n)
**General findings (n≥20[a])**			
Liquid blood in the organs or vessels	39		
Acute organ congestion	37[a]		
Severe general cyanosis	20[a]		
**Head/Neck (n≥19[a])**			
Brain edema	19[a]		
Petechiae in white matter of brain	6[a]		
Globus pallidus degeneration	1		
Cyanosis of the pharyngeal mucosa	1		
Cyanosis of the laryngeal mucosa	1		
Congestive bleeding in the tonsils	1		
Bleeding in the oblique neck muscles	1		
Bleeding in the pharyngeal mucosa	1		
Bleeding in the petrous bone		1	
Bleeding into the skull		1	
Subarachnoid hemorrhage		1	
Bleeding in the brain		1	
Skull base fracture		1	
**Face (n≥6[b])**			
Petechial hemorrhages in the conjunctiva	6[b]		
Bleeding of the conjunctiva	2		
Bleeding of the oral mucosa	1		
Facial congestion	1[b]		
Bleeding of the tongue		2	
Comminuted fracture of the skull		1	
Contusion of the temporal muscle		1	
Middle-ear bleeding		1	
Rupture of both eardrums		1	
**Thorax (n≥37[b])**			
Petechial hemorrhages of the pleura	13[b]		
Petechial hemorrhages of the pericardium	10[b]		
Petechial hemorrhages of the tracheal mucosa	3[b]		
Petechial hemorrhages of the lungs	1		
Dilation of the right ventricle	24		
Dilation of the left ventricle	4		
Dilation of the right atrium	1		
Extreme contraction of left ventricle	10		
Acute pulmonary emphysema	4		
Lung inflation	2		
Pulmonary edema or congestion	22		
Inflammatory edema	1		
Hypostasis of both lungs	1		
Bleeding in the epicardium/subepicardium	9		
Subserous bleeding	1		
Pleural or subpleural bleeding	4		
Subpleural contusions	1		

Bleeding in the thymus	1		
Massive aspiration pneumonia	1		
Aspiration of gastric contents or aspiration to peripheral bronchi	3		
Froth in the trachea and bronchi	6		
Inhalation of snow and water	1		
Tension pneumothorax		1	
Pulmonary bleeding		10	
Rib fractures		3	
Fat embolism		1	
Bleeding in the posterior mediastinum		1	
Esophageal hemorrhage		1	
**Abdomen (n≥9[a])**			
Petechiae of gastric mucosa	11[a]		
Petechial bleeding of mucosa in the small intestine	1		
Gastric mucosa bleeding	5		
Duodenal mucosa bleeding	1		
Inflation in the middle upper abdomen	1		
Congestion of pancreas	8[a]		
Hemorrhages of the pancreas	4[a]		
Bleeding in the spleen		1	
Perisplenic blood clot		1	
**Extremities/Pelvis (n=3)**			
Hip dislocation		1	
Knee dislocation		1	
Comminuted fracture of pelvis		1	
Comminuted fracture of leg		1	
Leg fracture		1	
**Skin (n≥23[a])**			
Petechial hemorrhages	3[a]		
Skin lacerations		3	
Skin abrasions		13	
Facial contusion		2	
Stab wound (lower extremity)		1	
Laceration of the perineum		1	
Contusion/laceration of the scalp		1	
Ecchymoses		2	
Subcutaneous bleeding		1	
Bleeding of he scalp		2	
Scalp hematoma		1	
Frostbite			2
**Other (n=4)**			
Petechial hemorrhages of the mucosa and under the serousmembranes (site not reported)	4		

[a] Refers to potentially overestimated cases; the exact number could not be determined because these findings were reported collectively for asphyxia-related deaths (n=18) and combined asphyxia-trauma-related deaths (n=6). Petechiae of the gastric mucosa were attributed to asphyxia, as the author [53] explicitly stated that none of the victims had Wischnewski spots, which are typically associated with hypothermia.

[b] Refers to potentially overestimated cases; the exact number could not be determined because these findings from Stalsberg were reported collectively for asphyxia-related deaths (n=10) and combined asphyxia-trauma-related deaths (n=2) [50].
