# Supplementary material for: Causes of death and types of injuries of avalanche fatalities based on forensic data: a scoping review
Source: Resusc Plus. 2025 Sep 13;26:101101. doi: 10.1016/j.resplu.2025.101101 (PMC12506530; doi:10.1016/j.resplu.2025.101101)
Supplement: Supplementary Data 3 [file mmc3.pdf]

Appendix C. Autopsy findings for the nine trauma-related deaths documented in seven studies [3,32,41,45,53,55,57].

Findings by anatomical regions	Number of victims (n)
**Head/Neck (n=8)**	
Fatal isolated cervical spine fracture with dislocation	2
Basilar skull fracture	2
Closed head injury	1
Fatal atlanto-occipital dislocation	1
Skull fracture	1
Brain hematoma	1
Splitting of the petrous bone	1
Skull, pia, and 4th ventricle bleeding	1
Fatal brain injury	1
**Face (n=1)**	
Extensive impression fracture of the face	1
Nasal bone fracture	1
Upper jaw fracture	1
Middle ear bleeding	1
**Thorax (n=4)**	
Rib fractures	3
Pulmonary contusion	2
Diaphragm rupture	1
Transected aorta (at the ligamentum anteriosum)	1
**Abdomen (n=1)**	
Subserous liver bleeding	1
**Extremities/Pelvis (n=1)**	
Upper extremity fracture	1
Pelvis fracture	1
**Skin (n=3)**	
Skin abrasions (face, upper/lower extremities)	2
Laceration and crush wounds (forehead, cheek, occiput)	1
Skin abrasions and lacerations (scalp, face)	1
