# Supplementary material for: Causes of death and types of injuries of avalanche fatalities based on forensic data: a scoping review
Source: Resusc Plus. 2025 Sep 13;26:101101. doi: 10.1016/j.resplu.2025.101101 (PMC12506530; doi:10.1016/j.resplu.2025.101101)
Supplement: Supplementary Data 4 [file mmc4.pdf]

**Appendix D. Autopsy findings for the nine combined asphyxia/trauma-related deaths documented in three studies [50,53,57].**

| Findings by anatomical regions (number of victims) | Lesions/injuries associated with asphyxia (n) | Lesions/injuries attributed to trauma (n) |
|---|---|---|
| **General findings (n≥6[a])** | | |
| Acute organ congestion | 6[a] | |
| Severe general cyanosis | 6[a] | |
| **Head/Neck (n≥3[a])** | | |
| Brain edema | 8[a] | |
| Petechiae of white matter of the brain | 6[a] | |
| Brain contusion | | 1 |
| Skull fracture | | 1 |
| **Face (n≥2[b])** | | |
| Facial congestion | 1[b] | |
| Petechial hemorrhages in conjunctiva | 2[b] | |
| **Thorax (n≥8[b])** | | |
| Petechial hemorrhages of the pleura | 2[b] | |
| Petechial hemorrhages of the pericardium | 2[b] | |
| Petechial hemorrhages of the tracheal mucosa | 2[b] | |
| Pulmonary edema | 2 | |
| Dilation of right ventricle | 2 | |
| Extreme contraction of left ventricle | 2 | |
| Thoracic deceleration injuries | | 1 |
| Rib fracture | | 1 |
| Pulmonary contusion | | 2 |
| Pulmonary parenchymal tear | | 2 |
| Hemothorax | | 3 |
| Thoracic vertebral fracture with spinal cord transection | | 2 |
| **Abdomen (n≥1[a])** | | |
| Petechiae of the stomach mucosa | 6[a] | |
| Congestion of pancreas | 6[a] | |
| Pancreatic hemorrhage | 4[a] | |
| Multiple liver fractures | | 1 |
| **Skin (n≥1[a])** | | |
| Petechial hemorrhages | 2[a] | |
| Skin abrasions | | 1 |
| **Other (n=2)** | | |
| Petechial hemorrhages of the mucosa and under the serous membranes (site not reported) | 1 | |
| Spinal trauma with spinal cord contusion | | 1 |

[a] Refers to potentially overestimated cases; the exact number could not be determined because these findings were reported collectively for asphyxia-related deaths (n=18) and combined asphyxia-trauma-related deaths (n=6) [53].

[b] Refers to potentially overestimated cases; the exact number could not be determined as these findings were reported collectively for asphyxia-related deaths (n=10) and combined asphyxia-trauma-related deaths (n=2) [50].
