# Supplementary material for: Causes of death and types of injuries of avalanche fatalities based on forensic data: a scoping review
Source: Resusc Plus. 2025 Sep 13;26:101101. doi: 10.1016/j.resplu.2025.101101 (PMC12506530; doi:10.1016/j.resplu.2025.101101)
Supplement: Supplementary Data 5 [file mmc5.pdf]

Appendix E. Autopsy findings for the five combined asphyxia/hypothermia-related deaths documented in three studies [32,38,57]. Lesions/injuries attributed to trauma were not reported as being or contributing to the cause of death on the original studies.

**Findings by anatomical regions**<br><br>**(number of victims)**	**Lesions/injuries associated with asphyxia**<br>**(n)**	**Lesions/injuries attributed to trauma**<br>**(n)**	**Lesions/injuries attributed to cold or general hypothermia**<br>**(n)**
**General findings (n=2)**			
Acute organ congestion	1		
Severe general cyanosis	1		
Liquid blood in the organs	1		
Liquid blood in the vessels	2		
**Head/Neck (n=1)**			
Brain edema	1		
**Face (n=1)**			
Cloudy corneas			1
**Thorax (n=2)**			
Dilation of the right ventricle	1		
Acute pulmonary emphysema	1		
Lung inflation	1		
Pulmonary edema	1		
Pleural or subpleural petechiae	1		
Bleeding of the diaphragm	1		
**Abdomen (n=3)**			
Bleeding of the anterior longitudinal ligament of spine, at promontory		1	
Wischnewski spots of the stomach			3
**Skin (n=2)**			
Hematoma		1	
Frostbite			1
**Other (n=2)**			
Petechial hemorrhages of the mucosa and under the serous membranes (site not reported)	2		
