# Supplementary material for: Causes of death and types of injuries of avalanche fatalities based on forensic data: a scoping review
Source: Resusc Plus. 2025 Sep 13;26:101101. doi: 10.1016/j.resplu.2025.101101 (PMC12506530; doi:10.1016/j.resplu.2025.101101)
Supplement: Supplementary Data 6 [file mmc6.pdf]

Appendix F. Pathology and histopathology findings for 48 avalanche fatalities documented in five studies [34,38,43,50,53].

Findings by organs (number of victims)	Lesions/injuries associated with asphyxia (n)	Lesions/injuries attributed to trauma (n)	Lesions/injuries attributed to cold or general hypothermia (n)
**Brain (n=1)**			
Ischemic damage to the cerebellum and neutrophilic edema	1		
**Heart (n≥19[a])**			
Interstitial edema	10		
Congestion	7		
Contraction band necrosis	6		
Marked interstitial edema with neutrophilic infiltration	1		
Increased proportion of white blood cells (some sections of the small vessels)	1		
Fatty degeneration (myocardial cells)			2
**Lungs (n=48)**			
Serosanguineous edema	26		
Focal intraalveolar hemorrhages	19		
Vascular congestion	15		
Intraalveolar edema	13		
Alveolar rupture	12		
Pulmonary congestion	12		
Acute emphysema	7		
Microscopic aspiration (gastric contents)	4		
Interstitial hemorrhages	3		
Mixed edema	2		
Hydroaeric edema	1		
Focal atelectasis	1		
Large platelet aggregates and accumulation of white blood cells (isolated pulmonary vessels)	1		
**Stomach (n=1)**			
Superficial gastric mucosal hemorrhages	1		
**Pancreas (n=15)**			
Absence of vacuoles			8
Few vacuoles			3
**Liver (n≥27[a])**			
Parenchymal congestion	24		
Hematic parenchymal dissociation		3	
**Spleen (n=17)**			
Vascular congestion	17		
**Kidneys (n≥18[a])**			
Congestion	17		
Hematic parenchymal suffusion with neutrophilic infiltration	1		
Tubular fatty degeneration			9

[a] Refers to potentially overestimated cases; the exact number could not be determined because these findings were not reported case-by-case [53].
